# Small Extracellular Vesicles in Neurodegenerative Disease: Emerging Roles in Pathogenesis, Biomarker Discovery, and Therapy

**DOI:** 10.3390/ijms26157246

**Published:** 2025-07-26

**Authors:** Mousumi Ghosh, Amir-Hossein Bayat, Damien D. Pearse

**Affiliations:** 1 The Miami Project to Cure Paralysis, University of Miami Miller School of Medicine, Miami, FL 33136, USA; axm8710@med.miami.edu (A.-H.B.); dpearse@med.miami.edu (D.D.P.); 2 The Department of Neurological Surgery, University of Miami Miller School of Medicine, Miami, FL 33136, USA; 3 The Neuroscience Program, University of Miami Miller School of Medicine, Miami, FL 33136, USA; 4 The Interdisciplinary Stem Cell Institute, University of Miami Miller School of Medicine, Miami, FL 33136, USA

**Keywords:** small extracellular vesicles, neurodegenerative diseases, intercellular communication, biomarkers, therapeutic delivery, liquid biopsy

## Abstract

Neurodegenerative diseases (NDDs) such as Alzheimer’s, Parkinson’s, ALS, and Huntington’s pose a growing global challenge due to their complex pathobiology and aging demographics. Once considered as cellular debris, small extracellular vesicles (sEVs) are now recognized as active mediators of intercellular signaling in NDD progression. These nanovesicles (~30–150 nm), capable of crossing the blood–brain barrier, carry pathological proteins, RNAs, and lipids, facilitating the spread of toxic species like Aβ, tau, TDP-43, and α-synuclein. sEVs are increasingly recognized as valuable diagnostic tools, outperforming traditional CSF biomarkers in early detection and disease monitoring. On the therapeutic front, engineered sEVs offer a promising platform for CNS-targeted delivery of siRNAs, CRISPR tools, and neuroprotective agents, demonstrating efficacy in preclinical models. However, translational hurdles persist, including standardization, scalability, and regulatory alignment. Promising solutions are emerging, such as CRISPR-based barcoding, which enables high-resolution tracking of vesicle biodistribution; AI-guided analytics to enhance quality control; and coordinated regulatory efforts by the FDA, EMA, and ISEV aimed at unifying identity and purity criteria under forthcoming Minimal Information for Studies of Extracellular Vesicles (MISEV) guidelines. This review critically examines the mechanistic roles, diagnostic potential, and therapeutic applications of sEVs in NDDs, and outlines key strategies for clinical translation.

## 1. Introduction

Neurodegenerative diseases (NDDs), including Alzheimer’s disease (AD), Parkinson’s disease (PD), amyotrophic lateral sclerosis (ALS), Huntington’s disease (HD), and related disorders, affect more than 50 million people worldwide and are poised to triple by 2050 as populations age [1,2,3]. Despite their diverse clinical phenotypes, NDDs share convergent hallmarks: selective neuronal loss, glial activation, and the accumulation of misfolded proteins such as amyloid-β, hyper-phosphorylated tau, α-synuclein, and TDP-43. The classic cell-autonomous paradigm of neurodegeneration is increasingly being replaced by systems-level models that emphasize the trans-synaptic and extracellular propagation of pathogenic proteins through interconnected neuronal and glial networks, as well as crosstalk with peripheral immune pathways [4]. A major driver of this paradigm shift is the discovery that small extracellular vesicles (sEVs) can transport toxic proteins, lipids, and nucleic acids between cells, thereby amplifying pathology while simultaneously providing a molecular “snapshot” of ongoing disease processes [5].

sEVs are 30–150 nm, double-membraned nanovesicles generated by the endosomal pathway and released via multivesicular body fusion or outward budding. Once dismissed as mere debris and labeled “exosomes,” sEVs are now recognized as potent transporters of proteins, lipids, and nucleic acids, distinct from larger microvesicles and apoptotic bodies [6]. First identified in reticulocytes in 1983 and formally termed “exosomes” in 1987 [7,8], their immunological function was firmly established when Raposo and colleagues demonstrated that B-cell-derived sEVs could present antigens to T cells [9]. Over the past decade, interest in sEVs in the context of NDD has expanded dramatically, with PubMed citations linking “exosome AND neurodegenerative” increasing over 30-fold, reflecting their rising significance in basic science, biomarker discovery, and therapeutic development [10].

## 2. Emerging Roles of sEVs in Neurodegeneration

### 2.1. Biogenesis and Molecular Cargo

The formation of sEVs is orchestrated through multiple interconnected pathways, including the endosomal sorting complexes required for transport (ESCRT)-dependent mechanism, ESCRT-independent routes involving lipid raft domains, and scaffolding by tetraspanins such as CD9, CD63, and CD81 [11]. Cargo selection is a highly regulated process influenced by post-translational modifications like ubiquitylation and sumoylation, as well as the action of RNA-binding proteins, which facilitate the selective packaging of proteins, lipids, and nucleic acids. This specificity underlies the emergence of disease-relevant signatures, for example, the enrichment of specific microRNAs like miR-9, miR-29, miR-135, and miR-186, which play a crucial role in Aβ generation [12,13] in AD, or phosphorylated Rab GTPases in PD linked to LRRK2 [13,14,15]. Encapsulated within a lipid bilayer, sEVs protect labile molecular contents from enzymatic degradation, allowing them to mediate stable, long-range communication both within the central nervous system (CNS) and between the CNS and peripheral tissues [16].

### 2.2. Crossing the Blood–Brain Barrier

sEVs possess a unique capacity to cross the blood–brain barrier (BBB) in both directions, distinguishing them from most synthetic nanocarriers. This bidirectional transport occurs through multiple mechanisms, including receptor-mediated transcytosis, adsorptive endocytosis, and trafficking via immune cells such as monocytes and macrophages, a phenomenon often referred to as “cellular hitchhiking” [17,18]. sEVs of CNS origin have been successfully detected in peripheral biofluids such as plasma, saliva, and urine, enabling the development of non-invasive liquid biopsies that reflect ongoing neuropathological processes. These vesicles offer considerable promise for early disease detection, longitudinal monitoring, and personalized therapeutic interventions in neurodegenerative disorders [19] while creating new opportunities for early diagnosis and real-time therapeutic monitoring [20].

### 2.3. Vectors of Pathology

**Scheme 1:**
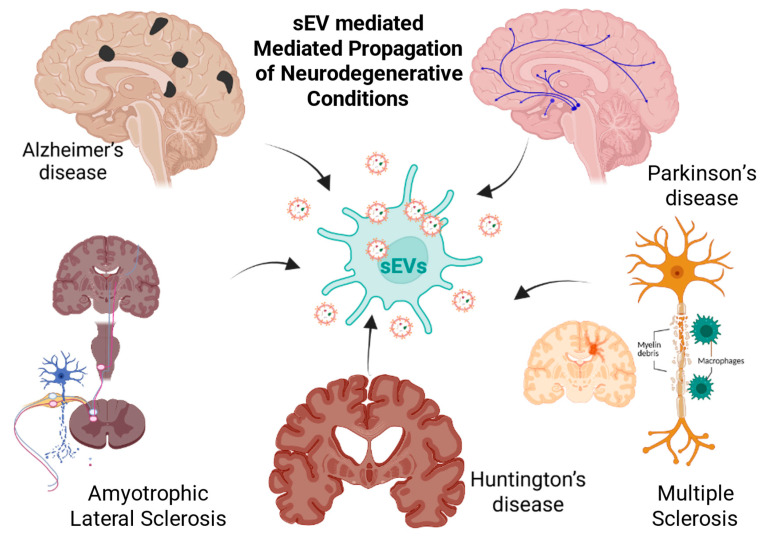
Role of sEVs in the spread and progression of NDDs. Pathogenic sEVs released in the CNS can transport misfolded proteins, inflammatory mediators, and nucleic acids that seed or amplify disease-specific lesions. The Scheme highlights five major disorders: Alzheimer’s disease (amyloid-β/tau spread), Parkinson’s disease (α-synuclein propagation), amyotrophic lateral sclerosis (motor neuron degeneration), Huntington’s disease (mutant huntingtin dissemination), and multiple sclerosis (immune-mediated demyelination), illustrating how sEVs contribute to the pathological spread and progression of these neurological conditions. (The scheme was created with BioRender.com. (accessed on 22 July 2025)).

Compelling evidence now supports the role of sEVs as vectors for the intercellular transmission of misfolded proteins, contributing to the prion-like spread of pathology in various NDDs [21,22] as shown in Scheme 1. Neuron-derived sEVs carrying oligomeric amyloid-β (Aβ) or hyperphosphorylated tau have been shown to enhance the deposition of amyloid plaques and neurofibrillary tangles in AD models [23]. Emerging studies support the existence of a dynamic microbiome-gut-brain axis, wherein microbial-derived metabolites and sEVs can traverse physiological barriers and influence neurodevelopment and neurodegeneration, as demonstrated in a gut-brain axis microfluidic model using human-iPSC-derived neurons, highlighting their potential as modulators of neural growth, synaptic plasticity, and CNS function [23]. There are reports of neuron-derived vesicles laden with oligomeric Aβ or tau seeds accelerating plaque and tangle deposition, while α-synuclein-rich gut sEVs ascend the vagus nerve to initiate PD-like changes in the substantia nigra. In ALS, sEVs secreted by motor neurons have been found to carry pathogenic species such as mutant superoxide dismutase 1 (SOD1) and TAR DNA-binding protein 43 (TDP-43), which elicit neurotoxic responses in astrocytes and microglia, fostering a feed-forward loop of neuroinflammation, thereby amplifying neuroinflammation through a self-reinforcing cycle [23]. These observations align with the emerging concept of sEV-mediated “connectomic spread” in NDDs [4,24].

### 2.4. Liquid Biopsy Potential

Recent technological advancements in immunoaffinity capture techniques targeting CNS-specific markers such as L1CAM and GLAST, along with innovations in microfluidics and single-vesicle omics, have significantly enhanced the ability to isolate and profile brain-derived sEVs from peripheral biofluids [25,26,27]. These brain-enriched sEVs (BEVs) carry disease-relevant molecular cargo, allowing for high-accuracy diagnostic applications. For instance, combined profiling of Aβ42, phosphorylated tau-181 (p-tau181), synaptophysin, and microRNA-21 within BEVs yields over 90% sensitivity and specificity in identifying prodromal AD [28]. Moreover, BEV-associated biomarkers such as neurofilament light chain (NfL) and phosphorylated TDP-43 have demonstrated superior performance over their plasma counterparts in staging and tracking progression in ALS [29]. Ongoing large-scale, longitudinal studies are underway to validate these sEV signatures and establish reference ranges across the aging spectrum [5,25,29].

### 2.5. Therapeutic Promise

sEVs offer a compelling platform for CNS drug delivery due to their inherent biocompatibility, low immunogenicity, and capacity for surface engineering and cargo customization [11,30]. Advanced bioengineering approaches such as the incorporation of rabies virus glycoprotein (RVG) peptides for neuron-specific targeting and CRISPR-Cas9 or siRNA loading for gene modulation have enabled sEVs to successfully deliver therapeutic payloads across the BBB, mitigating neuropathology in preclinical models of AD and PD [31,32,33]. These vesicles have been used to transport small molecules, nucleic acids, and genome-editing components with high target specificity and minimal off-target effects. Moreover, early-phase clinical trials utilizing mesenchymal stem cell (MSC)-derived sEVs have reported favorable safety profiles and preliminary signs of therapeutic efficacy in neurodegenerative and inflammatory CNS conditions [34,35,36].

### 2.6. Rationale and Scope of This Review

Amidst the accelerating pace of research into sEVs, there is a critical need for a comprehensive and up-to-date review of current knowledge. In this review, we aim to (i) delineate the molecular mechanisms governing sEV biogenesis and cargo sorting; (ii) critically examine experimental evidence supporting their role in the propagation of neurodegenerative pathology; (iii) assess the diagnostic and prognostic utility of sEV-based biomarkers in biofluids; (iv) explore emerging bioengineering strategies that leverage sEVs for therapeutic delivery across the BBB; and (v) identify key technical, translational, and regulatory hurdles that must be overcome to enable clinical application. Collectively, our objective is to provide a structured framework to harness the potential of sEVs to elucidate and ultimately intervene in the complex pathobiology of neurodegenerative disorders.

## 3. Determinants of sEV Composition in the CNS: Biogenesis, Cellular Origin, and Disease Modulation

### 3.1. Biogenesis and Molecular Determinants of Cargo Loading in sEVs

sEV biogenesis is not a random shedding process but a precisely tuned molecular event. Advances in single-vesicle sequencing, CRISPR-based labeling, and high-throughput proteomics have refined our ability to dissect sEV heterogeneity and decode their physiological relevance.

**Scheme 2:**
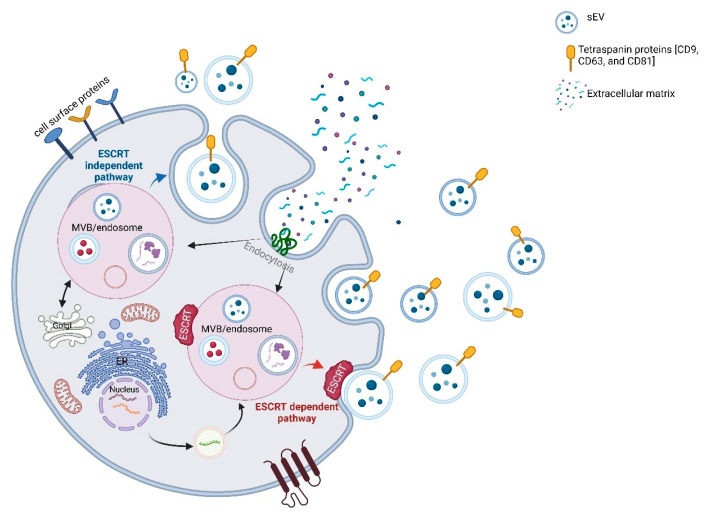
Biogenesis and cargo sorting mechanisms of sEVs. sEV formation begins with inward budding of the plasma membrane to generate early endosomes, which mature into multivesicular bodies (MVBs). Within MVBs, intraluminal vesicles (ILVs) are formed via two major pathways: the ESCRT-dependent pathway (red), involving endosomal sorting complexes required for transport, and the ESCRT-independent pathway (blue), driven by tetraspanins and specific lipids. Mature MVBs either fuse with lysosomes for degradation or with the plasma membrane to release ILVs as sEVs into the extracellular space. These vesicles carry proteins, RNAs, and lipids, and can be taken up by recipient cells via endocytosis, mediating intercellular communication. (The scheme was created with BioRender.com. (accessed on 22 July 2025)).

SEVs originate from the endosomal system and are released upon the fusion of multivesicular bodies (MVBs) with the plasma membrane. sEV biogenesis occurs through two major pathways: the ESCRT-dependent pathway and the ceramide-mediated ESCRT-independent pathway [37] as shown in Scheme 2. The ESCRT (endosomal sorting complex required for transport) machinery consists of four protein complexes (ESCRT-0, -I, -II, and -III) and accessory proteins such as ALIX and TSG101, which coordinate cargo recognition, membrane budding, and vesicle scission. Disruption of ESCRT components, such as CHMP2B, has been linked to familial frontotemporal dementia, underscoring the importance of this pathway in neurobiology [38]. In parallel, ESCRT-independent sEV formation is driven by ceramide generation via neutral sphingomyelinase 2 (nSMase2), promoting negative membrane curvature. This mechanism is particularly active in glial cells and can be pharmacologically inhibited using agents like GW4869 [39,40]. Tetraspanins such as CD9, CD63, and CD81 organize into specialized microdomains that scaffold vesicles budding independently of ESCRT components. Super-resolution microscopy has revealed that CD9 modulates the intracellular trafficking of CD63; depletion of CD9 enhances CD63-positive EV secretion, whereas CD9 overexpression suppresses it [41].

Cargo loading into sEVs is increasingly recognized as a highly regulated process rather than a passive reflection of cytoplasmic contents [42]. Protein inclusion relies on multiple signals, including ubiquitination (which facilitates ESCRT recognition), lipid raft partitioning, and tetraspanin-mediated clustering of membrane proteins such as MHC molecules and L1CAM [37]. Post-translational modifications like phosphorylation and sumoylation further influence cargo sorting under both physiological and pathological conditions, such as misfolded tau or TDP-43, particularly under pathological conditions like ALS or AD [43].

RNA cargo selection, encompassing miRNAs, mRNAs, and non-coding RNAs, is guided by RNA-binding proteins (RBPs) such as hnRNPA2B1, AGO2, and YBX1, which recognize short sequence motifs such as EXOmotifs and mediate vesicular packaging [44]. Disease states such as AD can alter this sorting process, resulting in enrichment of specific miRNAs (e.g., miR-34a, miR-125b, miR-146a) and lipids that may contribute to neuroinflammation and synaptic dysfunction [45,46].

Together, the biogenesis and selective cargo loading of sEVs reflect an intricate interplay between endosomal trafficking machinery, membrane dynamics, and molecular recognition systems. These properties not only enable sEVs to serve as intercellular messengers but also position them as potential biomarkers and therapeutic targets in a range of diseases.

### 3.2. Cell-Type-Specific sEV Profiles in the CNS

In the CNS, sEVs exhibit cell-type-specific molecular compositions that reflect their origin and functional specialization. Major CNS cell types, including neurons, astrocytes, microglia, and oligodendrocytes, release sEVs that contribute to both homeostatic signaling and disease-related processes. Neuronal sEVs are typically characterized by surface markers such as L1CAM and NCAM and are enriched with synaptic proteins including synaptophysin and postsynaptic density protein 95 (PSD-95), as well as neurotransmitter receptors and activity-regulated RNAs. The composition of neuronal sEVs is dynamically regulated by synaptic activity, brain-derived neurotrophic factor (BDNF) signaling, and cellular stressors such as excitotoxicity or oxidative damage [47,48,49,50]. Astrocyte-derived sEVs, often identified by glutamate-aspartate transporter (GLAST) and glial fibrillary acidic protein (GFAP), can transfer neuroprotective molecules including apolipoprotein E (ApoE) and glutamate transporters under physiological conditions. However, in pathological contexts, these vesicles may carry neurotoxic components such as ceramide, pro-inflammatory cytokines, and microRNAs (e.g., miR-21, miR-34a), contributing to neuroinflammation and synaptic dysfunction [51,52,53]. Microglial sEVs play critical roles in neuroimmune communication and synaptic remodeling. These vesicles contain immune-related molecules such as major histocompatibility complex (MHC) class II proteins, interleukin-1β (IL-1β), and pro-inflammatory microRNAs including miR-155, thereby mediating inflammatory responses and microglia–neuron crosstalk [54,55]. Oligodendrocyte-derived sEVs are enriched with myelin-associated proteins such as proteolipid protein (PLP) and myelin oligodendrocyte glycoprotein (MOG), along with glycolytic enzymes that support axonal metabolism [56]. These vesicles have been shown to promote axonal integrity and may exert neuroprotective effects in demyelinating diseases, including experimental models of multiple sclerosis (MS) [56]. Collectively, these distinct cell-specific sEVs profiles underscore the functional heterogeneity of sEVs in CNS intercellular signaling and highlight their potential as biomarkers and therapeutic targets in neurological disorders.

### 3.3. Influence of Stress and Disease on the Composition of sEVs

The molecular cargo of sEVs is dynamically regulated in response to cellular stress and pathological conditions, reflecting disease-specific changes in their proteomic and transcriptomic content. In AD, sEVs derived from cerebrospinal fluid (CSF) and plasma exhibit increased levels of amyloid-β (Aβ) peptides, phosphorylated tau (p-tau), and regulatory microRNAs such as miR-29a/b and miR-34a, which are implicated in amyloid genesis and synaptic dysfunction [28,57,58]. In PD, dopaminergic neurons subjected to genetic mutations (e.g., LRRK2) or environmental toxins release sEVs enriched in α-synuclein, phosphorylated LRRK2, and Rab10, indicating a role in disease propagation and intracellular trafficking defects [59,60]. In ALS and FTD models, sEVs derived from motor neurons and glial cells have been shown to carry SOD1, TDP-43, and cryptic RNAs associated with C9orf72 repeat expansions, contributing to RNA toxicity and neuroinflammation [51,59,61]. Similarly, in HD, the sEV cargo changes dynamically with motor activity and disease progression, including the presence of mutant huntingtin protein (mHTT), BDNF-modulating microRNAs, and altered synaptic mRNA transcripts, suggesting a role in neuroplasticity and disease modulation [62,63].

## 4. Physiological Functions of Small EVs in the Healthy CNS

A significant number of studies have convincingly demonstrated that sEVs are not simply a by-product of neural activity; they underpin routine “house-keeping” and long-range communication between cells across the CNS. Below, we elaborate on each major domain in which neuronal and glial nanovesicles shape circuit development and maintain adult neural homeostasis.

### 4.1. Neuronal sEVs: Tuning Synapse Formation, Maturation, and Plasticity

Neuronal sEVs are emerging as critical regulators of synaptic development, maturation, and plasticity. During early neurodevelopment, neurons secrete sEVs enriched in cell adhesion molecules such as NCAM-1 and L1CAM, Wnt family morphogens, and activity-regulated microRNAs [64]. Studies by Zhang et al. (2021) showed that neuronal sEVs enriched in HDAC2 mediate intercellular signaling that downregulates synaptic gene expression and modulates dendritic spine density during cortical development [65]. These vesicles function in a paracrine manner to guide axonal pathfinding, promote dendritic spine stabilization, and enhance filopodial outgrowth in recipient neurons, thereby facilitating synaptogenesis and circuit assembly. A recent systematic review synthesizing data from over 120 primary studies identified exosome-associated proteins including ephrin-B2, semaphorin-3A, and neuroligin-1 as key contributors to axon guidance and synaptic specificity, functioning in concert with classical chemotropic cues to refine callosal and topographic connectivity [66].

In the mature brain, neuronal sEVs continue to influence synaptic efficacy and plasticity in an activity-dependent manner. High-frequency neuronal firing induces glutamate release and postsynaptic Ca^2+^ influx, which in turn activates Rab35 and PIKfyve signaling pathways that govern vesicle trafficking and secretion. These activity-evoked sEVs are enriched in microRNAs such as miR-132, miR-134, and miR-212, which are internalized by neighboring postsynaptic compartments. Once delivered, these miRNAs suppress targets such as PTEN and LIMK1, resulting in increased dendritic spine size and elevated thresholds for long-term potentiation (LTP) [67]. This mechanism has been implicated in experience-dependent synaptic remodeling and is thought to underlie aspects of mood regulation and cognitive flexibility [68].

### 4.2. Microglial sEVs and Complement-Driven Synaptic Pruning

During postnatal development, microglia orchestrate synaptic refinement through complement-mediated pruning, selectively eliminating excess or weak synapses to sculpt neural circuits. Classical complement components C1q and C3 are deposited onto synapses, marking them for engulfment by microglial processes. Emerging evidence reveals that sEVs derived from microglia serve as a conduit for delivering C1q to neuronal targets. A recent study demonstrated that microglia are the predominant source of extracellular C1q in the brain. Once secreted, C1q associates with neuronal ribonucleoprotein granules, promoting their degradation and facilitating synapse elimination by licensing complement receptor interactions on microglia [69]. Beyond contact-dependent mechanisms, microglia also engage in remote, vesicle-mediated synaptic pruning. Pro-inflammatory activation induces the expression of the membrane-associated sialidase Neu3, which is incorporated into fusogenic microglial sEVs. Upon membrane fusion with presynaptic terminals, Neu3 removes terminal sialic acid residues, exposing galactose moieties that enhance complement deposition and subsequent pruning [70]. Functionally, blockade of the complement cascade or genetic ablation of C1q preserves inhibitory synapses following exposure to general anesthetics, indicating that this complement-sEV axis is both physiologically active and pharmacologically modifiable [71].

### 4.3. Oligodendrocyte-Derived sEVs: Metabolic Lifelines for Axons and Myelin Upkeep

In addition to facilitating saltatory conduction, myelinating oligodendrocytes play a crucial role in maintaining the metabolic integrity of long-projection axons by supplying energetic and antioxidant support. This is achieved through two primary mechanisms: (a) the transfer of metabolic substrates such as lactate and pyruvate via the monocarboxylate transporters MCT1 (on oligodendrocytes) and MCT2 (on axons), and (b) the release of sEVs enriched with glycolytic enzymes, antioxidant proteins, and the mitochondrial deacetylase SIRT2 [72]. Once internalized by neurons, SIRT2 deacetylates adenine nucleotide translocators ANT1 and ANT2, enhancing ADP-ATP exchange across the mitochondrial inner membrane and boosting oxidative phosphorylation efficiency. Impairment of this vesicle-mediated metabolic coupling leads to axonal swellings, impaired mitochondrial function, and conduction failure; conversely, supplementation with purified oligodendrocyte-derived sEVs restores axonal energy homeostasis and conduction fidelity [73].

This support system is dynamically regulated by neuronal activity. Action potential propagation triggers the release of glutamate into the periaxonal space, which in turn activates NMDA and AMPA receptors localized on oligodendrocyte inner processes. This receptor engagement elevates intracellular Ca^2+^ levels and activates the CaMKK-AMPK signaling cascade, linking neuronal firing to glucose uptake and the biogenesis of metabolically active exosomes [74]. Thus, oligodendrocyte sEV-mediated metabolic support is activity-dependent, preferentially targeting energetically demanding, high-frequency axons and aligning cellular bioenergetics with network dynamics.

### 4.4. Integrated Glia–Neuron Crosstalk and Myelin Maintenance

Astrocyte-derived sEVs close the metabolic loop by donating glycogen-derived pyruvate to oligodendrocytes and secreting factors (e.g., LIF) that promote myelin sheath stability, evidence that the three glial lineages cooperate through intersecting vesicle channels [75]. Collectively, these data reposition sEVs as pivotal arbiters of CNS physiology, balancing connectivity via microglial pruning, fine-tuning synaptic gain through neuronal miRNAs, and fueling the immense energetic load of myelinated tracts via oligodendrocyte cargo delivery. It is clear that even in the absence of disease, sEVs continually sculpt neural circuits, clear redundant synapses, and safeguard axonal metabolism functions that become maladaptive only when cargo composition or release kinetics go awry.

## 5. Small EVs in NDD Pathogenesis

sEVs act as vectors of pathogenic proteins and inflammatory signals and as dynamic biomarkers reflecting molecular changes. Recent mechanistic discoveries reveal novel therapeutic and diagnostic strategies across major neurodegenerative disorders.

### 5.1. Small EVs in Alzheimer’s Disease: Biomarker Signatures, Pathogenic Spread, and Therapeutic Modulation

**Scheme 3:**
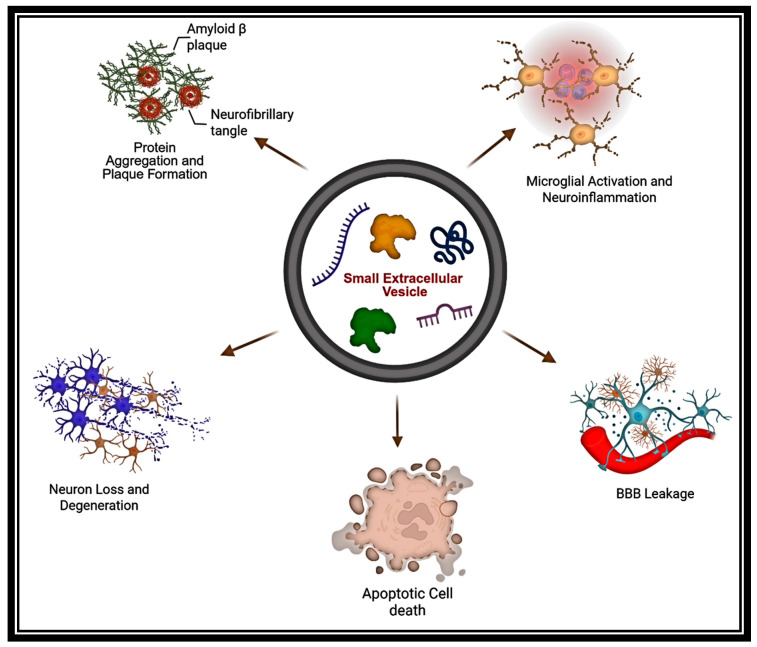
Role of sEVs in the propagation of NDDs. This illustration depicts how sEVs contribute to NDD progression by transporting neurotoxic cargo that promotes protein misfolding, neuroinflammation, BBB disruption, and synaptic dysfunction, thereby spreading pathology throughout the CNS. (The Scheme was created with BioRender.com. (accessed on 22 July 2025)).

sEVs play a critical role in AD by carrying pathogenic biomarkers, mediating the spread of tau and amyloid pathology, and offering new therapeutic targets as listed in Table 1. In this section, we highlight their emerging relevance in early detection, disease progression, and intervention strategies, as shown in Scheme 3.

**Cargo signatures that are predictive mRNA and inflammatory protein biomarkers:** Plasma-derived BEVs carry mRNAs such as *CADM1*, *GABRB3*, and *FOXJ3,* which, when used in machine learning algorithms, distinguish AD from preclinical stages and accurately predict age at onset (>90% accuracy) [57]. Complementary miRNA profiling confirms increased miR-34a and miR-125b, two regulators of dendritic spine stability and complement activation [46]. Recent studies have shown that EVs facilitate the propagation of inflammasome signaling within both the brain and heart in murine models of AD [76].

**Propagating the classical lesions and mediation of tau and amyloid propagation:** BEVs from APOE4 carriers carry tau filaments that facilitate trans-neuronal spread of tauopathy, a process enhanced by specific lipidomic cargo [77]. sEVs released from neurons that over-express GSK3β-phosphorylated tau transfer seed-competent tau to neighboring cells, accelerating tangle formation in human-iPSC-derived cortical cultures and wild-type mice [78]. Oligomeric Aβ, in turn, is loaded into astrocyte-derived sEVs, whose CD63-rich surface dramatically enhances neuronal uptake and toxicity; a recent study shows that catalpol + tetramethyl-pyrazine drives astrocytes to secrete CDK5-mRNA-positive sEVs that potentiate STAT3 signaling and axonal sprouting, underscoring how glial vesicles can flip from protective to harmful depending on cargo [79].

**Emerging therapeutic angles via sEV modulation.** Pharmacological inhibition of neutral sphingomyelinase 2 (nSMase2) has been shown to significantly reduce neuronal exosome secretion and decrease interstitial levels of amyloid-β (Aβ) and phosphorylated tau in 5xFAD AD mouse models. These findings have catalyzed the development of combination therapeutic strategies such as pairing nSMase2 inhibitors with anti-Aβ monoclonal antibodies, which are now advancing through preclinical pipelines as a means to synergistically enhance Aβ clearance and attenuate neurodegeneration [80].

### 5.2. Parkinson’s Disease (PD)

Similar to as in AD, sEVs have emerged as key mediators in PD, contributing to gut–brain signaling, biomarker discovery, and the development of novel therapeutic strategies.

**Gut-brain trafficking of α-synuclein:** Enteric-origin α-synuclein loaded into sEVs has been shown to be transmitted via the vagus nerve to the dorsal motor nucleus, thereby providing a physiological vector for Braak stage progression in PD models [81]. Exposure of rodents to rotenone, an environmental mitochondrial toxin, induces α-synuclein release from enteric neurons, and both surgical vagotomy and sEV tetraspanin blockade effectively impede its central propagation [82]. In LRRK2-G2019S mutation carriers, high-resolution phosphoproteomic profiling of urinary and plasma sEVs reveals elevated levels of phosphorylated Rab12 at Ser106; these levels rapidly decline after only one week of treatment with the LRRK2 inhibitor MLi-2, highlighting pSer106-Rab12 as a dynamic pharmacodynamic and biomarker read-out [83].

**Toward sEV-guided interventions:** A recent review has endorsed BEV panels comprising α-synuclein, DJ-1, and miRNA for early-stage diagnostic use, with superior performance to CSF assays [84]. Moreover, therapeutic strategies leveraging sEV-mediated delivery are advancing: ultrasound-mediated CRISPR interference targeting *SNCA* within sEVs achieves approximately 70% knockdown of α-synuclein and rescues nigral dopaminergic degeneration in A53T mouse models, while antisense oligonucleotide-loaded, RVG-tagged sEVs similarly mitigate pathology via RNA interference [33,85,86].

### 5.3. Amyotrophic Lateral Sclerosis (ALS)

Motor-neuron-derived sEVs facilitate intercellular propagation of misfolded TDP-43 and mutant SOD1, which are internalized by astrocytes and microglia, thereby intensifying non-cell-autonomous neuroinflammatory cascades in ALS models [87,88]. Quantitative assays show that plasma sEV-associated TDP-43 discriminates ALS from frontotemporal dementia and healthy controls with an area under the curve (AUC) > 0.9, and these levels correlate longitudinally with motor function decline over 12 months [89]. While serum NfL is an FDA-qualified biomarker, accumulating evidence indicates that BEV NfL increases earlier and more closely tracks corticospinal tract degeneration than serum NfL, suggesting substantial potential to reduce diagnostic latency, a finding emphasized in the 2024 EV-ALS biomarker compendium [90]. Therapeutically, pharmacological blockade of sEV biogenesis using GW4869 in SOD1-G93A mice attenuates weight loss and extends survival, and MSC-derived sEVs engineered to deliver anti-TDP-43 siRNA restore motor neuron electrophysiological function in human cervical spinal cord organoids. Both strategies are now approaching Investigational New Drug-enabling stages, underscoring the translational promise of targeting sEV-mediated mechanisms in ALS [91,92].

### 5.4. Multiple Sclerosis (MS)

sEVs are increasingly recognized for their dual roles in MS, both as mediators of pathology and as therapeutic vehicles. Pathologically, CNS-derived sEVs traverse the BBB and transfer pro-inflammatory cargo such as myelin antigens and microRNAs that activate peripheral immune cells and exacerbate CNS demyelination and neuroinflammation [93]. Conversely, MSC-derived sEVs exert potent immunomodulatory effects: in vitro, they shift microglia from pro-inflammatory to anti-inflammatory phenotypes, suppress Th17 differentiation, and reprogram T cells toward regulatory subsets, while in vivo, they promote remyelination and reduce clinical disability in experimental autoimmune encephalomyelitis (EAE) models.

Oligodendrocyte-derived sEVs have recently been shown to bridge central and peripheral immunity in MS. These vesicles carry myelin-associated peptides such as MBP, MOG, and PLP that are presented to antigen-presenting cells (APCs) in peripheral lymphoid tissues, thereby priming autoreactive T cells and contributing to CNS-targeted autoimmunity [94]. Clinically, plasma sEVs bearing the integrins CD29, CD31, and adhesion molecule CD44 increase during active demyelinating lesion formation, with elevated levels observable approximately four weeks before corresponding spikes in serum NfL, suggesting a predictive biomarker role for these sEV signatures [95]. These findings have catalyzed the development of sEV-based therapeutic approaches, with MSC-sEVs advancing into early-phase clinical investigation as a cell-free regenerative and immunomodulatory treatment for MS [96].

### 5.5. Huntington’s Disease (HD)

sEVs, have been increasingly implicated in the intercellular propagation of mHTT, representing a pathogenic mechanism in HD. In vitro and in vivo studies demonstrate that neurons and glial cells secrete mHTT species, both monomers and oligomers within sEVs, which are subsequently internalized by neighboring cells, contributing to non-cell-autonomous neuronal toxicity and neurodegeneration [97]. Notably, LC-MS and imaging analyses have identified mHTT, including full-length (~360 kDa) and ~70 kDa truncated forms, within circulating sEVs from HD patients and transgenic porcine models, indicating systemic release of pathogenic protein via vesicular export [98]. Furthermore, functional studies using human striatal neurons transplanted into mouse brains revealed that sEVs bearing mHTT elicit degeneration of host neurons, a process attenuated by pharmacological inhibition of secretion via sphingosine-1-phosphate receptor antagonists (e.g., FTY720) [99]. Cumulatively, these findings support a model wherein sEV-mediated transfer of mHTT contributes substantively to HD pathogenesis and reinforces the rationale for targeting sEV biogenesis or uptake as novel therapeutic avenues in HD.

Conversely, proteomic analyses of striatal sEVs demonstrate that motor skill training induces a remodeling of vesicle cargo, enriching sEVs with synaptic plasticity proteins such as PSD-95 and Homer1, as well as metabolic enzymes including GAPDH and LDH-B [62]. These training-induced sEVs mitigate mHTT toxicity in neuronal co-culture assays, suggesting that activity-dependent exosome release may contribute to the delayed disease onset observed with environmental enrichment [100]. Concurrent work implicates microglia-derived sEVs enriched in BDNF, specifically splice variant IV, in rescuing corticostriatal synapse loss in HD models, positioning glial sEVs as endogenous modifiers of circuit integrity [101].

### 5.6. sEVs in Chronic CNS Injury (Chronic SCI and TBI)

sEVs play a dual role in chronic CNS injury such as spinal cord injury (SCI) and traumatic brain injury (TBI), acting both as mediators of secondary neurodegeneration and as vehicles for tissue repair. Pathogenically, sEVs released from injured neural and glial cells carry pro-inflammatory cytokines, damage-associated molecular patterns (DAMPs), and microRNAs that activate microglia and infiltrating immune cells, exacerbating neuroinflammation, excitotoxicity, and neuronal apoptosis in distal brain regions [102].

It has been demonstrated in chronic SCI that sEVs act both as mediators of lasting neurodegenerative signaling and as vehicles for regenerative therapies. Pathogenically, long-term SCI alters tissue- and sex-specific sEV secretion: in chronic SCI models, circulating sEV profiles display sexual dimorphism and correlate with persistent neuroinflammatory markers and cognitive deficits, implicating systemic, sEV-mediated neurodegenerative crosstalk [103]. In parallel, injurious stimuli generate sEVs containing pro-inflammatory cytokines, microRNAs, or DAMPs, which propagate inflammation and neuronal apoptosis beyond the lesion site. Emerging evidence identifies miR-21 as a central modulator in SCI, where it influences key processes such as inflammation, cell death, and repair by regulating molecular targets like PTEN and PDCD4 and shaping post-injury signaling networks [104]. Therapeutically, MSC-derived sEVs have demonstrated potent neuroprotective and reparative capacities: in preclinical SCI models, MSC-sEVs reduce inflammation, inhibit oxidative stress, promote blood–spinal cord barrier restoration, and support axonal regrowth and motor recovery [105,106]. These findings underscore that while aberrant sEV signaling sustains neurodegeneration in chronic SCI, engineered stem-cell-derived sEVs offer a promising cell-free strategy for mitigating pathology and promoting neurological repair.

Similarly, in TBI, circulating sEVs containing neuronal proteins and miRNAs serve as robust biomarkers for injury severity, metabolic dysfunction, and neuroinflammatory states [107]. Studies have reported that sEVs play a central role in propagating inflammasome signaling after CNS injury by carrying inflammasome components such as ASC and caspase-1 [108]. Conversely, sEV-mediated delivery of siRNA can effectively suppress inflammasome activation, offering a promising therapeutic strategy for reducing neuroinflammation and secondary damage following CNS trauma [108]. Recent studies showed that serum sEVs serve as potential biomarkers of TBI. Acute serum exosomal miR-206 and miR-549a-3p predict six-month cognitive outcome; MSC-sEVs curb early neuro-inflammation and white matter loss [109,110]. 

**Table 1:** Roles of sEVs in neurodegenerative and traumatic CNS pathology: functional insights and cargo profiles.

Disease/Condition	sEV Function/Role	Key sEV Cargo	References
Alzheimer’s Disease	Spread of Aβ and tau; modulate microglial inflammation and synaptic dysfunction	Aβ, phosphorylated tau, miR-132-5p, miR-193b, miR-125b, miR-485-5p, miR-23a, miR-125b	[46,111]
Parkinson’s Disease	Transmit misfolded α-synuclein; influence dopaminergic neuron survival	α-synuclein, DJ-1, LRRK2, Let-7f-5p and miR-125a-5p, miR-10b-5p and miR-151a-3p, miR-24, miR-195, POU3F3	[112,113]
Amyotrophic Lateral Sclerosis	Transfer TDP-43, SOD1; promote motor neuron degeneration	TDP-43, SOD1, FUS, miR-124, miR-146a-5p; miR-199a-3p; miR-151a-3p; miR-151a-5p; miR-199a-5p, MiR-151a-5p	[114,115]
Huntington’s Disease	Disseminate mHTT aggregates; modulate synaptic toxicity and inflammation	mutant huntingtin (mHTT), miR-124	[116]
Multiple Sclerosis	Amplify inflammatory responses; alter T cell and BBB function	miR-155, CD29, CD31, inflammatory cytokines	[95,117,118]
Chronic Spinal Cord Injury	Carry pro-inflammatory miRNAs and cytokines; inhibit regeneration, sustain gliosis	miR-21, cytokines (TNF-α, IL-1β), GFAP	[104]
Traumatic Brain Injury	Alter neurovascular signaling; deliver injury-responsive miRNAs and DAMPs	miR-873, HSP70, NLRP3, mitochondrial DNA	[119]

Conversely, therapeutic strategies are leveraging the regenerative capabilities of sEVs. Neural-stem-cell-derived sEVs enhance motor recovery, attenuate inflammatory microglial activation, reduce neuronal apoptosis, and promote autophagy in chronic SCI models [120]. Moreover, MSC-derived sEVs loaded with anti-inflammatory microRNAs or neurotrophic factors mitigate oxidative stress, support BBB repair, and improve functional outcomes in both SCI and TBI paradigms [121]. Intranasal delivery of human MSC-derived sEVs suppress NLRP3 inflammasome activation and downstream p38/MAPK signaling following TBI, thereby mitigating chronic neurological dysfunction [122]. Similarly, sEVs derived from Schwann cells (SCs), the principal myelinating cells of the peripheral nervous system, have demonstrated therapeutic potential in experimental models of both SCI and TBI, highlighting their promise as future candidates for neuroregenerative interventions [123,124,125]. Collectively, these findings highlight that dysregulated, injury-derived sEVs contribute to chronic neurodegeneration, whereas engineered sEVs represent promising cell-free interventions now progressing toward translational studies in repairing chronic CNS damage. sEVs can also provide versatile therapeutic tools to redefine our understanding of disease propagation, enabling minimally invasive biomarker discovery, and paving the way for next-generation, precision-targeted interventions.

## 6. Small EV Biomarkers: Current Landscape, Technical Hurdles, and Clinical Promise

sEVs represent a transformative advance in NDD diagnostics because they can traverse the BBB intact and carry molecular signatures reflective of the CNS tissue from which they are derived [126]. Immunocapturing these sEVs via cell-type-specific surface markers, such as neuronal L1CAM/CD171, astroglial GLAST, or oligodendroglial MOG, enables a 100-fold enrichment for CNS-derived cargo relative to bulk plasma, allowing ultrasensitive detection via techniques like digital ELISA (Simoa), bead-based flow cytometry, and low-input RNA sequencing [127]. These assays often surpass the performance of matched serum or CSF tests and can detect pathology well ahead of time, before symptom onset. In AD, neuron-derived BEVs routinely show elevated Aβ_42_ and phosphorylated tau (p-tau181), alongside reduced synaptic proteins such as synaptophysin, with additional increases in miR-21, miR-34a, and miR-125b, capturing neuroinflammatory and synaptic loss signals. In large meta-analyses, a four-analyte BEV panel (Aβ_42_, p-tau181, synaptophysin, and miR-21) discriminated prodromal AD from controls with 92% accuracy, and p-tau181 levels predicted hippocampal atrophy while synaptophysin tracked cognitive decline [128]. For PD, neuron-derived BEV α-synuclein seed amplification detected misfolded protein up to a decade pre-diagnosis, and sEV phospho-Rab12^Ser106^ in LRRK2-G2019S carriers reflected response to LRRK2 inhibitor therapy [83,129]. In a recent study of ALS, plasma sEV TDP-43 levels were significantly elevated in ALS compared to healthy controls and other NDD, with AUC values ≥ 0.94 distinguishing ALS from controls. These sEV TDP-43 levels correlated more strongly with disease severity measures, including ALS Functional Rating Scale-Revised (ALS-FRS–R) scores, and cognitive performance than serum NfL alone [89]. Additional applications include determining relapses in MS (miR-155/150 ± CD29/CD31) [130], sEV-associated GFAP as a circulating biomarker of TBI severity correlating with CT-positive lesions post-trauma (EV-GFAP + UCH-L1) [131], and molecular subtyping in frontotemporal dementia via sEV-TDP-43 and tau isoforms [89]. Despite these advances, technical challenges persist, chiefly in ensuring capture specificity, such as avoiding L1CAM expression from peripheral sources via dual epitope strategies [132], controlling pre-analytical variability (centrifugation, freeze–thaw cycles) [133], advancing quantification methods while overcoming critical analytical hurdles with novel emerging platforms such as digital ELISAs for microfluidic proteomics to enable improved sEV quantification [134], and developing standardized reference materials. Looking forward, single-vesicle multi-omics promises to integrate genotype (e.g., APOE, C9orf72), while longitudinal digital phenotyping paired with minimally invasive sEV sampling may enable real-time, adaptive biomarker monitoring [135,136]. In summary, when applied under rigorous MISEV-2023 standards and combined with ultra-sensitive multi-analyte detection, sEV assays stand poised to transition from exploratory research tools to clinically validated diagnostics across NDDs [136].

## 7. Small EVs as Therapeutic Platforms for CNS Disorders: Engineering, Delivery, and Clinical Translation

**Scheme 4:**
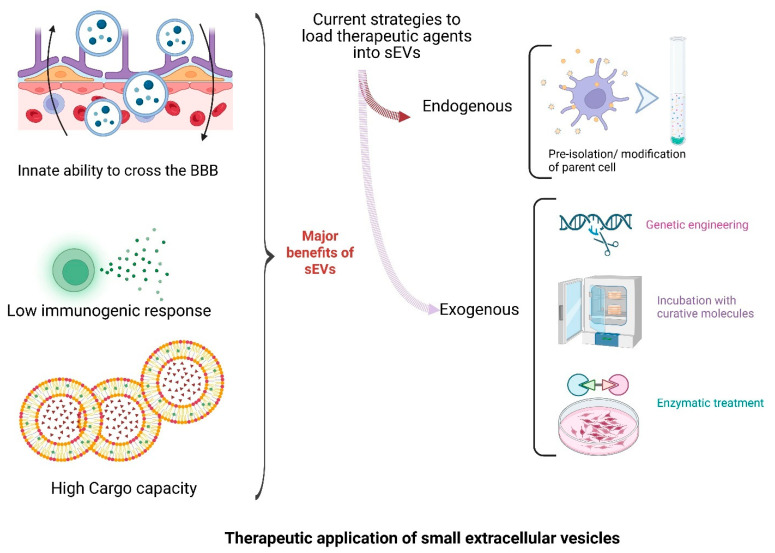
Advancing neurodegenerative treatments through sEV-based approaches. sEVs offer significant therapeutic potential due to their innate ability to cross the blood–brain barrier, low immunogenicity, and high cargo capacity. Therapeutic payloads can be introduced via endogenous (parent cell modification) or exogenous (genetic, chemical, or enzymatic) strategies, enabling versatile and targeted delivery platforms for CNS disorders. (The scheme was created with BioRender.com. (accessed on 22 July 2025)).

sEVs have emerged as promising nanocarriers for CNS therapeutics, primarily due to their innate ability to cross the BBB, low immunogenic profile, and versatile cargo capacity [137] (see Scheme 4). These vesicles leverage endogenous biological pathways, including receptor-mediated transcytosis and immune evasion mechanisms, attributed to host-derived membrane proteins and negligible endotoxin content, allowing for efficient brain-wide delivery of pharmacological payloads at significantly reduced doses [138,139]. Their endogenous origin allows for the encapsulation of small molecules, nucleic acids, proteins, and CRISPR ribonucleoproteins, making them ideal platforms for gene and drug delivery across neural tissues [140].

Various strategies have been developed to load therapeutic agents into sEVs (see Scheme 4). Passive loading through co-incubation or permeabilization permits the incorporation of lipophilic drugs such as rapamycin and curcumin [141,142]. Active methods like sonication or saponin permeabilization further enhance loading efficiency [143]. For example, rapamycin-loaded sEVs restored tight-junction integrity and reduced neuroinflammation in a murine model of BBB dysfunction at one-tenth the conventional dose [144]. Co-loading of polyphenols such as curcumin and methylene blue in MSC-derived sEVs has demonstrated additive effects in reducing Aβ and tau pathology while improving cognitive outcomes in AD mouse models [145]. Advanced microfluidic platforms and electroporation allow for efficient encapsulation of RNA-based cargoes, such as siRNA and CRISPR components, although yield losses remain a limiting factor, spurring innovations like nanoporative chips with up to 80% retention efficiency [146,147].

**Surface engineering of sEVs facilitates cell-specific targeting**. The fusion of rabies virus glycoprotein (RVG) peptide with Lamp2b, a sEV membrane protein, has been shown to enhance neuronal uptake, enabling delivery of siRNAs targeting BACE1 and α-synuclein, thereby reversing behavioral deficits in NDD models [148,149]. Similarly, engineering donor cells to express anti-CD19 single-chain variable fragments (scFv) resulted in sEVs capable of crossing the BBB and delivering methotrexate to CNS lymphoma sites, achieving therapeutic effects with a 70% lower systemic dose and minimized toxicity [150]. Chemical ligation strategies, such as click-chemistry with RGD or transferrin, are advancing toward GMP-compliant, non-genetically modified vesicle coatings [151]. The therapeutic use of sEVs has extended to nucleic acid delivery and gene editing. For instance, siRNA-loaded vesicles targeting LRRK2 significantly reduced kinase hyperactivity and neuroinflammation in PD models [152]. Similarly, RVG-functionalized sEVs transporting mRNA for BDNF and nerve growth factor (NGF) demonstrated restoration of hippocampal neurogenesis following ischemic injury [153].

Stem-cell-derived sEVs, particularly from MSCs, are naturally enriched with miRNAs such as miR-124-3p and miR-146a-5p, key regulators of synaptic plasticity and NF-κB signaling. Augmenting donor cells to overexpress these miRNAs yields more potent vesicles; for example, miR-124-sEVs restored dendritic spine density and improved cognitive function in 3×Tg-AD mice, while miR-146a-sEVs attenuated astrocyte-mediated cytokine release and improved outcomes in CNS injury models [154,155,156,157]. Infusion of sEVs derived from bone marrow MSCs as a therapeutic approach for ALS reported no serious adverse events [158]. Subsequently, a first human case report demonstrated safety and potential clinical stabilization associated with intravenous infusions of allogeneic SCEVs in a patient with advanced ALS, offering a novel therapeutic avenue targeting this neurological condition [159].

Manufacturing and regulatory challenges remain major bottlenecks in clinical translation. Hollow-fiber bioreactors now enable scalable production of GMP-grade sEVs, yielding >10^11^ particles/mL while meeting sterility and particle-to-protein ratio requirements [160]. However, no sEV-based therapeutic has received FDA approval as a standalone product marketed for therapeutic use. Regulatory agencies classify sEVs as biologics, requiring IND-level characterization, including identity, purity, potency, and safety data. In conclusion, sEVs represent a clinically promising, biologically compatible platform for targeted CNS therapy. Continued progress in molecular engineering, scalable biomanufacturing, and regulatory harmonization will be essential for realizing their full clinical potential.

## 8. Comparative Assessment of Diverse Carrier Platforms for CNS Drug Delivery

Delivering therapeutics to the CNS remains a major hurdle because drugs must cross the BBB while retaining bioactivity and safety In addition to cell-derived small extracellular vesicles other drug delivery platforms that are currently under investigation include synthetic liposomes/lipid nanoparticles, biodegradable polymeric nanoparticles, and viral vectors, each offering a unique blend of payload capacity, immunogenicity, manufacturing complexity, and regulatory standing. Table 2 presents these platforms side-by-side, summarizing their principal strengths and limitations to support informed selection or combination for brain-targeted drug-development efforts.

## 9. Challenges and Future Directions

The clinical translation of sEVs as diagnostics and therapeutics now hinges more on overcoming methodological limitations than on resolving biological uncertainties. Key barriers lie in several interrelated domains: isolation, precision and reliability, scalable manufacturing, safety and immunogenicity, evolving regulatory landscapes, and analytic innovation. Addressing these bottlenecks through coordinated technical and policy advances will be critical to advancing the field in the coming years.

### 9.1. Standardization of Isolation and Characterization

The isolation of sEVs remains a major source of variability. Common methods such as differential ultracentrifugation, polymer-based precipitation, size-exclusion chromatography, immune-affinity capture, asymmetric-flow field-flow fractionation, and microfluidic or acoustofluidic platforms enrich for overlapping but distinct vesicle populations. Multicenter studies demonstrate that particle-to-protein ratios can vary by more than two orders of magnitude for a single sample, introducing profound inconsistency in biomarker discovery and therapeutic potency [171,172]. The 2023 update of the MISEV guidelines provides a comprehensive, expert-driven update on current methodologies, best practices, and emerging techniques for sEV production, isolation, characterization, and in vivo study across diverse biological sources [133].

### 9.2. Scaling Manufacturing Without Compromising Identity

**Scheme 5:**
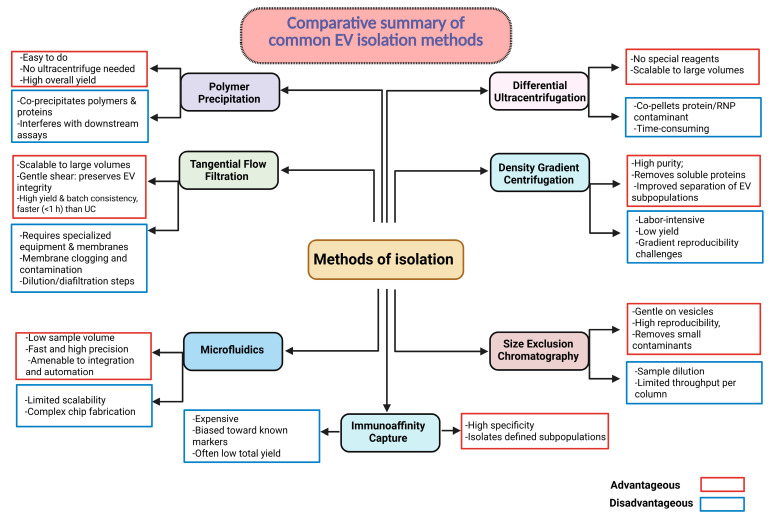
sEV isolation methods: advantages vs. limitations. Each method has distinct trade-offs in yield, purity, throughput, cost, and effort; selecting or strategically combining methods is dependent on specific downstream application.

Purifying sEVs hinges on separating intact nanoscale vesicles from cells, protein complexes, and polymers without compromising vesicle integrity or bioactivity. Core workflows fall into six major categories: (i) differential ultracentrifugation, which exploits size- and mass-dependent sedimentation; (ii) density gradient centrifugation, isolating vesicles by buoyant density; (iii) size-exclusion chromatography (SEC), where porous resins partition particles by hydrodynamic radius; (iv) immunoaffinity capture that targets surface antigens for sub-population specificity; (v) polyethylene glycol (PEG) precipitation, which concentrates vesicles via volume exclusion; and (vi) tangential flow filtration (TFF), a scalable cross-flow membrane process that concentrates sEVs and performs buffer exchange under gentle shear. Traditional cell culture flasks yield high-purity but low-volume sEV preparations, typically <10^10^ particles per liter. Hollow-fiber bioreactors (HFBs) have demonstrated sustained production and increased yields by over 30-fold while maintaining consistent physical and functional properties [173,174]. TFF is replacing ultracentrifugation to prevent shear-induced membrane disruption, and beta-stage perfusion-based systems with in-line diafiltration promise further efficiency and cost reductions [175]. A critical challenge in sEV production is achieving consistent bioactivity and cargo composition, which must be rigorously controlled to meet regulatory standards for safety, potency, and reproducibility. The lack of standardized potency assays remains a major regulatory bottleneck. As no single isolation method optimally balances purity, yield, scalability, and cost, combinatorial or orthogonal strategies are often necessary to align with MISEV guidelines and downstream clinical requirements [176]. Scheme 5 summarizes currently employed sEV purification techniques.

### 9.3. Immunogenicity and Biodistribution Concerns

**Scheme 6:**
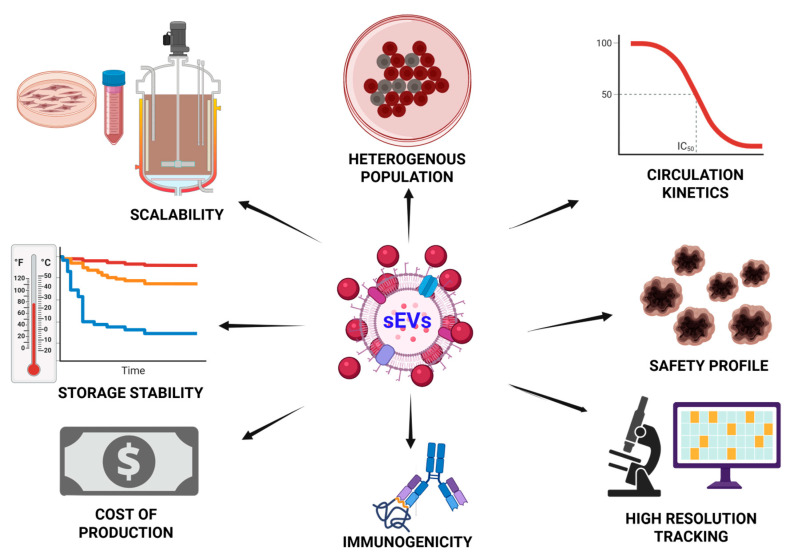
Key challenges in the therapeutic application of sEVs. Therapeutic application of sEVs requires comprehensive evaluation of multiple factors including source cell type, production scalability, drug loading efficiency, potency, immune response, biodistribution, stability, cost, and analytical characterization. Optimizing these parameters is essential for successful clinical translation of sEV-based therapeutics. (The scheme was created with BioRender.com. (accessed on 22 July 2025)).

Although autologous sEVs are generally well tolerated and exhibit low immunogenicity, engineered or allogeneic vesicles can activate innate immune pathways depending on their cargo and surface composition. For instance, tumor-derived sEVs enriched in miR-21 have been shown to activate Toll-like receptors 7 and 8 (TLR7/8), inducing proinflammatory cytokines such as IL-6 and TNF-α, and promoting cachexia in murine cancer models [10,177]. Similarly, neuronal sEVs containing double-stranded RNA can engage TLR3, amplifying neuroinflammation, particularly in the context of opioid exposure [178]. To counter these effects, preclinical strategies under investigation include CRISPR-mediated depletion of immunostimulatory RNA, partial PEGylation of vesicle membranes to reduce immune detection, and CD47 overexpression to inhibit macrophage-mediated clearance [179,180]. Accurate tracking of sEV biodistribution remains a critical challenge, as conventional lipophilic dyes like DiR and PKH may form micelles, leading to artifactual overestimation of CNS uptake [181,182]. CRISPR-based barcoding methods, such as guiding RNA fusion to the sEV marker CD63, enable high-resolution tracking of individual sEV subtypes through deep sequencing [183]. These tools have revealed distinct in vivo biodistribution patterns between CD9^+^ and CD63^+^ sEVs, challenging prior assumptions and highlighting the need for refined, biologically specific tracking approaches in translational sEV research. If these scientific and regulatory challenges can be met, sEVs may soon transition from bench curiosities to front-line nanotherapeutics for complex diseases such as neurodegeneration (See Scheme 6).

## 10. Conclusions

In just over a decade, sEVs have emerged as central tools in NDD research, functioning as vectors of pathogenic proteins, diagnostic biomarkers, and therapeutic delivery vehicles. They play key roles in disease propagation, early detection, and targeted treatment. Recent advances include brain-enriched diagnostic panels, engineered therapeutic sEVs, and scalable high-precision manufacturing technologies. While challenges remain such as standardization and in vivo tracking, technological innovations and evolving regulatory frameworks are rapidly addressing them. With ongoing scientific advancements, sEVs are emerging as a pivotal modality in NDD management, ushering in a new era of precision nano-neurotechnology that seamlessly integrates diagnosis and therapy into a single, powerful platform, allowing earlier detection, targeted intervention, and potential disease modification.

## Figures and Tables

**Table 2:** Comparison of CNS-targeted drug delivery platforms.

Feature	sEVs	Liposomes/LNPs	Polymeric NPs	Viral Vectors
Typical Size	30–150 nm	20–200 nm (up to 1 µm)	50–300 nm (tunable 20 nm–1 µm)	AAV: ~25 nm; lentivirus: ~100 nm
Biocompatibility	Very high (endogenous origin)	High (PEGylation improves circulation)	Moderate–high	Moderate–high (protein capsid; genomic risk)
Immunogenicity	Low	Moderate (anti-PEG antibodies)	Moderate–high (surface-charge-dependent)	High (neutralizing antibodies, innate/adaptive responses)
Cargo Capacity	Moderate, multiple protein molecules (~10^3^); fewer nucleic-acid copies per vesicle	Very high (both hydrophilic and lipophilic drugs)	Moderate (<20% *w*/*w*, burst-release common)	Limited (AAV: ~4.7 kb; lenti: ~8–10 kb)
BBB Penetration	Modest; via transcytosis, hitchhiking	Poor intrinsic BBB crossing; requires ligands, FUS, or chemical disruption	Poor (<5%) unless actively targeted	Excellent when using neurotropic pseudotypes (e.g., AAV9, PHP.eB)
Targeting Strategies	Intrinsic tropism: surface engineering (RVG-Lamp2b), click-chemistry	Ligand Conjugation	PEGylation, surface modifications	Native tropism; pseudotyping envelope engineering, tissue-specific promoters
Scalability/Regulatory Status	Moderate; pilot GMP, heterogeneity challenges, several IND MSC-EV trials, one single patient SC-EV clinical investigation, no FDA approvals	High and well established, multiple FDA-approved	High; scalable, FDA-approved PLGA depots; no CNS-targeted PNPs yet	CMC more stringent, strict QA/QC, several FDA-approved gene therapies
Key Advantages	Biocompatible with native tropism and low immunogenic, can cross BBB, flexible loading (RNA, proteins, CRISPR), repeat dosing, surface easily engineered	Very high drug-loading flexibility, well-established GMP and analytics, robust regulatory track record	Tunable composition and degradation, controlled drug release profiles, broad polymer versatility	High transduction efficiency, long-term expression from single dose, extensive serotype availability
Limitations	Heterogeneity; lack of robust potency assays; isolation variability	Rapid clearance, stability issues; manufacturing complexity	Polymer degradation, burst release, scale-up variability	Immunogenicity, insertional risk, size limits, manufacturing cost
References	[10,138,161]	[162,163,164]	[165,166,167]	[168,169,170]

## Data Availability

Not applicable.

## Abbreviations

The following abbreviations are used in this manuscript:

ANT1	Adenine nucleotide translocators
AD	Alzheimer’s disease
AMPK	AMP-activated protein kinase
ALS	Amyotrophic lateral sclerosis
APCs	Antigen-presenting cells
ApoE	Apolipoprotein E
AGO2	Argonaute 2
BBB	Blood–brain barrier
BDNF	Brain-derived neurotrophic factor
CaMKK	Calcium/calmodulin-dependent protein kinase kinase
CADM1	Cell adhesion molecule 1
CNS	Central nervous system
CSF	Cerebrospinal fluid
C9orf72	Chromosome 9 open reading frame 72
CRISPR	Clustered Regularly Interspaced Short Palindromic Repeats
CDK5	Cyclin-dependent kinase 5
DAMPs	Damage-associated molecular patterns
ESCRT	Endosomal sorting complexes required for transport
ELISA	Enzyme-Linked Immunosorbent Assay
EMA	European Medicines Agency
EAE	Experimental autoimmune encephalomyelitis
EV	Extracellular vesicle
FDA	Food and Drug Administration
FOXJ3	Forkhead box O3
GABRB3	Gamma-aminobutyric acid; receptor subunit beta-3
GFAP	Glial fibrillary acidic protein
GLAST	Glutamate/aspartate transporter
GAPDH	Glyceraldehyde-3-phosphate dehydrogenase
GSK3β	Glycogen synthase kinase 3 beta
hnRNPA2B1	Heterogeneous nuclear ribonucleoprotein A2/B1
HDAC2	Histone deacetylase 2
HD	Huntington’s disease
IL-1β	Interleukin-1β
ISEV	International Society for Extracellular Vesicles
IND	Investigational New Drug
L1CAM	L1 cell adhesion molecule
L1CAM/CD171	L1 cell adhesion molecule
LRRK2	Leucine-rich repeat kinase 2
LIMK1	LIM domain kinase 1
Lamp2b	Lysosomal-associated membrane protein 2
LNP	Lipid nanoparticle
MHC	Major histocompatibility complex
MSC	Mesenchymal stem cell
MSC-EVs	Mesenchymal-stem-cell-derived EVs
MISEV-2023	Minimal Information for Studies of Extracellular Vesicles guidelines 2023
MCT1	Monocarboxylate transporters
MS	Multiple sclerosis
MVBs	Multivesicular bodies
mHTT	Mutant huntingtin protein
MBP	Myelin basic protein
MOG	Myelin oligodendrocyte glycoprotein
NGF	Nerve growth factor
NDDs	Neurodegenerative diseases
NFL	Neurofilament light
nSMase2	Neutral sphingomyelinase 2
NMDA	N-methyl-D-aspartate
NP	Nanoparticle
PD	Parkinson’s disease
PTEN	Phosphatase and tensin
PEGylation	Polyethylene glycol
PSD-95	Postsynaptic density protein 95
PDCD4	Programmed cell death protein 4
PLP	Proteolipid protein
RVG	Rabies virus glycoprotein
RBPs	RNA-binding proteins
SCEVs	Schwann cell (SC)-derived exosomal vesicles
STAT3	Signal transducer and activator of transcription 3
sEVs	Small extracellular vesicles
SCI	Spinal cord injury
SOD1	Superoxide dismutase 1
TDP-43	TAR DNA-binding protein 43
TLR7/8	Toll-like receptors 7 and 8
TBI	Traumatic brain injury
TNF-α	Tumor necrosis factor alpha
YBX1	Y-box binding protein 1
AMPA	α-amino-3-hydroxy-5-methyl-4-isoxazolepropionic acid
